# Editorial: The future of perceptual illusions: from phenomenology to neuroscience, vol II

**DOI:** 10.3389/fnhum.2023.1214050

**Published:** 2023-06-27

**Authors:** Bangio Pinna, Adam Reeves

**Affiliations:** ^1^Department of Architecture, Design and Planning, University of Sassari at Alghero, Sassari, Italy; ^2^Department of Psychology, Northeastern University, Boston, MA, United States

**Keywords:** Frontiers, cognitive neuroscience, Research Topic on illusions, vision, illusions, definition, prediction, phenomena

Can illusions provide insights into the operations of a perceptual system? If so, do they also cast light on the operations of the brain, and do they predict behavior—do false impressions cause erroneous actions? After all, illusions might be artistic but represent only unimportant failures of a system that otherwise extracts needed information from the visual environment. These questions raise large issues, several of them addressed by the papers in this Research Topic.

Rogers (2022) asks “What is an illusion?” taking exception to Gregory's definition of illusions as “*departures from reality*.” Reality is hard to define and most “illusions” are either mistakenly labeled, or are simple consequences of “how our perceptual systems work,” or reflect impoverished stimuli. Labeling the Ames room and Adelson's checker shadow as “illusions” is mistaken; both are facsimiles of real stimuli that produce similar patterns of light on the eye. An example of “how the system works” is metamerism; color mixture is a true “departure from reality” but is not classified as “illusory,” its cause (trichromacy) being known. “Illusions” due to impoverished stimuli have been discussed since J. J. Gibson, and need no outing here.

Tyler accepts (and expands on) Gregory's classification of illusions, but also objects to Gregory's claim that illusions depart from reality as assessed by “simple measurements with rulers, photometers, clocks, and so on.” Instead, illusions occur when the perceiver can directly compare the illusory percept with the “ground truth” as revealed by direct observation. This neatly eliminates Rogers' paradox that metamerism would count as “illusory” for Gregory. The Ames room remains illusory, as people's heights are directly verifiable outside it. Tyler's phenomenalistic definition of illusion may prove more useful than Gregory's.

Blakeslee and McCourt study illusory brightness induction from adjacent and remote backgrounds, arguing they produce brightness *contrast* effects of the same or opposite polarity, depending on the luminance polarity of these regions relative to target patch luminance. Target brightness is determined by the relative strength and direction of brightness contrast from the adjacent and remote backgrounds, but, importantly, there is no evidence for brightness assimilation.

Kimura (2023) demonstrates with flanking bar stimuli that color spreading occurs in both the “neon color” condition, in which the colors appear transparent, and when the colors appear opaque, as in the watercolor effect, depending on the luminance relations between the center, the bars, and the background. Importantly, the rules that transparent and opaque illusory colors follow differ: Color spreading is transparent when the luminance relations are appropriate for perceptual scission, whereas opaque spreading is found when they are not. Thus, the visual mechanisms that control them likely also differ.

Laeng et al. present a white field with a large, blurry, central *black* oval surrounded by a large number of small sharp black ovals. For 80% of observers, the central black oval appears to expand toward them, and as it does, the apparent brightness of the display decreases. In Kitaoka's “Ashai” illusion, a central *white* blurry region, surrounded by darker ovals, appears brighter than the white field. The authors report that pupil diameters are 4.8 mm for black holes and 3.4 mm for white holes, an astounding difference of 1.4 mm due entirely to illusory brightness differences.

Mruczek et al. (2022) find that the Ebbinghaus, and Corridor illusions, which increase when the stimulus is moved, do so especially when the context changes in size at the same time as the target moves. Target motion actually eliminated the usual Ponzo illusion unless both context and target changed, when the illusion was restored or increased. Several interacting factors, including size constancy mechanisms, may ultimately account for these complex results.

Bachmann (2022) reports an illusion in which a schematic face appears merry or worried depending on how one interprets its parts, specifically the mouth and chin, which illustrates a novel visual ambiguity, not between objects (“duck/rabbit”), nor in figure/ground relationships (“vase/faces”), but within the same object seen entirely as figure. He argues that this offers a new way for disentangling higher and lower-level factors in perception.

Kaneno and Ashida discuss the sense of body ownership during the rubber hand illusion. They hypothesized that a smiling facial expression would elicit a stronger rubber hand illusion, compared to a neutral or disgusted face. Subjects elicited these expressions by varying the position of a wooden chopstick held in the mouth. The proprioceptive drift of the real hand toward the fake one, a motoric index of the illusion, is enhanced when the subjects displayed a disgusted compared to a happy face, but the subjective reports of the illusion were *not* affected, a novel disassociation.

Wilson et al. presented Neker cubes for 1 s, followed by blank ISIs of 400 ms, so they could synchronize stimulus onset to the band-pass filtered multi-electrode EEG, and find that perceptual reversals of the cube are predicted almost one second before they appear by a change in ERGs generated mostly in the right para-hippocampal place area. The reversals seem spontaneous, but are predictable ahead of time.

Sugihara and Pinna presented 2D wireframe pictures that have different 3-D interpretations based on either rectangularity or symmetry, but, unlike standard images such as the Necker cube, not both. They find that rectangularity generally trumps symmetry, and this rather remarkably helps explain some illusory visual deformations of specific solid objects when the viewpoint is moved continuously.

Zavagno et al. report that Mona Lisa's image appears to look directly at an observer when the picture is viewed from 4 m or more away, but becomes more ambiguous as the image is brought closer in, with increasing numbers of people judging her gaze to be diverted. Critically, at 0.5 m, the eyes alone, when cropped out of the entire image, were still judged to be smiling - almost as much as was the entire face - even though they appeared to be diverted. My ‘take' (not that of the authors) is that Leonardo captured an intrinsic expression, one of self-satisfaction, rather than a communicative facial gesture.

In conclusion, it seems clear that the term “illusion” covers too much ground for any over-arching theory of them to be plausible. Rather, illusions may either reflect systematic error in the visual system, not pruned out by evolution, or result from the proper working of a visual mechanism selected by evolution but tested with reduced stimuli. Both cases are of interest to those who wish to probe the working of the visual brain. A final point is that some illusions do “depart from reality” as determined by photometric (Blakeslee and McCourt; Laeng et al.) and colorimetric (Kimura, 2023) measurements, and seem none the less illusory for all that.

## Author contributions

BP and AR wrote the editorial. All authors contributed to the article and approved the submitted version.
